# Real-world data analysis of the risk of Fournier’s gangrene in patients using sodium-glucose cotransporter 2 inhibitors (SGLT2i)

**DOI:** 10.3389/fphar.2025.1643866

**Published:** 2025-12-10

**Authors:** Moritz Hertel, Hanno Jaoulak, Max Heiland, Susanne Nahles, Robert Preissner, Saskia Preissner

**Affiliations:** 1 Department Oral and Maxillofacial Surgery, Charité – Universitätsmedizin Berlin, Corporate Member of Freie Universität Berlin, Humboldt-Universität zu Berlin, and Berlin Institute of Health, Berlin, Germany; 2 Department of Nephrology and Intensive Care, St. Joseph Hospital, Berlin, Germany; 3 Institute of Physiology and Science-IT, Charité – Universitätsmedizin Berlin, Corporate Member of Freie Universität Berlin, Humboldt-Universität zu Berlin, and Berlin Institute of Health, Berlin, Germany

**Keywords:** fournier’s gangrene, SGLT2 inhibitors, real-world data pharmacovigilance, necrotizing fasciitis, diabetes complications, adverse drug events

## Abstract

**Background:**

The aim of the present matched cohort study was to assess if patients using sodium-glucose cotransporter 2 inhibitors (SGLT2i) show an augmented risk of Fournier’s gangrene (FG) compared to subjects not using SGLT2i.

**Methods:**

Retrospective data were retrieved from the TriNetX database, whereby patients using SGLT2i and individuals not using SGLT2i were assigned to cohorts I and II, respectively. After matching for age, gender distribution, and prevalence of diabetes mellitus, human immunodeficiency virus infection, alcohol abuse, liver diseases, and use of immune suppressant, a risk analysis was carried out, and risk ratio (RR) and odds ratio (OR) were determined. The outcome was defined as diagnosis of FG.

**Results:**

Before matching, cohorts I and II accounted for 192,245 and 7,810,583 subjects. After matching, 192,245 patients (40.5% females and 59.5% males; mean age 67.5 ± 12.7 years) remained per cohort. Among the cohorts I and II, 186 and 312 individuals were diagnosed with FG. The according risks of 0.1% and 0.2% were significantly different (p > 0.001; Log-Rank test). RR and OR were 0.539 (95% CI 0.447-0.650) and 0.539 (95% CI 0.446-0.650).

**Conclusion:**

The present study found a significantly lower risk of FG in patients using SGLT2i in comparison with subjects not using SGLT2i. Due to specific limitations that come alongside with the applied study design, this result needs to be interpreted most cautiously. As the cohorts were matched for the frequency of diabetes mellitus (68.9% of the cohorts I and II) this risk factor for FG might have been mitigated among the individuals in cohort I due to the use of SGLT2i, which may in turn explain the abovementioned finding.

## 1 Introduction

Sodium-glucose cotransporter 2 inhibitors (SGLT2i, empagliflozin, canagliflozin, dapagliflozin, ertugliflozin, ipragliflozin, luseogliflozin, sotagliflozin, togogliflozin) block the reuptake of glucose and sodium in the proximal renal tubules resulting in glycosuria (Heerspink et al., 2016). In addition, pleiotropiceffects in terms of enhanced natriuresis, intravascular volume contraction, and altered intra-renal hemodynamics beneficially influence blood pressure, body weight, and albuminuria (Heerspink et al., 2016; Heerspink et al., 2018). Large placebo-controlled studies revealed that SGLT2i prevent atherosclerotic cardiovascular disease, heart failure (HF), and chronic kidney disease (CKD) in subjects suffering from type 2 diabetes mellitus (T2DM) (Nuffield Department of Population Health Renal Studies G and Consortium SiM-AC-RTSGLT2 inhibitor Meta-Analysis Cardio-Renal Trialists' Consortium, 2022). They furthermore lower the risk of CKD progression and kidney failure among patients with T2DM and proteinuric kidney disease (Staplin et al., 2021; Neuen et al., 2019). SGLT2i were also found to reduce the risk of hospitalization as well as mortality in individuals with acute heart failure (AHF) with or without diabetes and irrespective of ejection fraction status (Staplin et al., 2021; Zannad et al., 2020; Heidenreich et al., 2022).

Despite the abovementioned favorable effects, adverse events can occur when using SGLT2i. Meta-analyses of Toyama et al. and Qiu et al. reported significantly augmented risks of genital infections (risk ratio [RR] 2.86, 95% confidence interval [CI] 2.00-4.10 and RR 3.75, 95% CI 3.00-4.67) (Toyama et al., 2019; Qiu et al., 2021), diabetic ketoacidosis (RR 2.57, 95% CI 1.53-4.3) (Qiu et al., 2021), and volume depletion (RR 1.14, 95% CI 1.05-1.24) (Qiu et al., 2021). The authors also found increased trends in the risks of fracture (RR 1.07, 95% CI 0.99-1.16), amputation (RR 1.21, 95% CI 0.97-1.51), and infections of the urinary tract (RR 1.07, 95% CI 0.99-1.15) (Qiu et al., 2021). Among genital infections, Fournier’s gangrene (FG) is a rare, but life-threatening necrotizing fasciitis of the perineum. Between March 2013 and May 2018 twelve cases (seven males and five females) of FG among individuals using SGLT2i in the United States were reported to the MedWatch program of the Food and Drug Administration (FDA). As a consequence, the FDA released a Drug Safety Communication on SGLT2i use and a potential risk of FG in 2018 and repeatedly in 2022 (Administration USFaD, 2025). Nevertheless, supporting evidence is widely lacking. A meta-analysis performed by Silverii et al. involving 84 clinical trials and a total number of 69,573 patients did not find an augmented risk of FG among subjects using SGLT2i (Mantel-Heanzel odds ratio [MH-OR] 0.41, 95% CI 0.09-1.82) (Silverii et al., 2020). A post-hoc pooled analysis investigating the safety of SGLT2i performed by Watada et al. included 2,895 patients from five clinical studies, whereby no cases of FG were noted (Watada et al., 2020). Fisher et al. set up a matched cohort study analyzing real-world data to compare the risk of urinary sepsis in individuals using SGLT2i (n = 208,244) with patients using dipeptidyl peptidase-4 inhibitors (DPP4i), whereby cases of FG were recorded as secondary outcome. The incidence of FG did not differ significantly between both cohorts (in SGLT2i cohort: 0.08 per 1,000 person-years, 95% CI 0.05-0.13), at least within the mean follow-up period of 0.9 years (Fisher et al., 2020).

However, an association of SGLT2i administration and an augmented risk of FG remains a matter of ongoing debate. Accordingly, the present study aimed to compare the risk of FG among individuals using SGLT2i in comparison with patients not using SGLT2i by analyzing real-world data. The aim of the present study was to investigate the risk of Fournier’s gangrene in individuals using SGLT2 inhibitors compared to patients not using these agents, based on real-world data. This research was motivated by existing safety concerns, conflicting evidence from previous studies, and the rarity of this severe condition, which limits the feasibility of randomized controlled trials.

## 2 Materials and methods

### 2.1 Data assessment, inclusion, and exclusion criteria

A real-world data set provided by the TriNetX Global Health Research Network (TriNetX, Cambridge, Massachusetts, United States) was analyzed to test the hypothesis. TriNetX is a real-world database allowing access to medical records of over 275 million patients from more than 120 healthcare organizations (HCOs) in 19 countries. To reduce the influence of potential confounding factors related to extreme body composition, such as cachexia or obesity, the analysis was restricted to individuals with a body mass index (BMI) between 19.00 and 30.00 kg/m^2^. This range was chosen to focus on patients with normal to moderately elevated BMI, for whom the baseline risk of infections or altered drug metabolism is less likely to be systematically different. To reduce potential confounding due to local trauma, infections, or altered anatomical conditions, individuals who had undergone operative procedures on the genitourinary system within the 12 months prior to the index event were excluded. These procedures included, but were not limited to, circumcision, urinary catheterization, transurethral prostate surgery, orchiectomy, or surgical interventions involving the perineum or external genitalia. While the study population was not restricted to patients with specific SGLT2i indications, the vast majority of included individuals had diagnoses of type 2 diabetes, heart failure, or chronic kidney disease, reflecting current clinical use patterns. This was verified during the data extraction process. Clinical conditions were identified based on ICD-10 codes recorded prior to the index event. Specifically, diabetes mellitus was defined by documented diagnosis codes (E10–E14), irrespective of medication status. HIV infection was identified via codes B20–B24. Alcohol abuse was defined using ICD-10 codes F10.1–F10.9 and K70, reflecting both behavioral and organ-specific consequences. Liver diseases included both alcoholic (e.g., K70) and non-alcoholic liver conditions (e.g., K76), without further differentiation due to coding limitations. Use of immunosuppressants was defined by prescription records and included systemic corticosteroids (e.g., prednisolone), calcineurin inhibitors (e.g., tacrolimus, cyclosporine), antimetabolites (e.g., azathioprine, mycophenolate), and biologics (e.g., TNF-α inhibitors), depending on availability in the TriNetX drug classification. The obtained individuals were consecutively assigned to cohorts I (continuous use of SGLT2i) and II (patients who had never been treated with SGLT2i).

### 2.2 Propensity score matching

To mitigate confounder bias via the propensity score and to replicate randomized conditions as closely as possible, one-to-one matching for age, gender distribution, and frequencies of diabetes mellitus, human immunodeficiency virus (HIV) infection, alcohol abuse, liver diseases, and use of immune suppressants was conducted using a nearest neighbor greedy matching algorithm with a caliper of 0.25 times the standard deviation. Variables such as chronic kidney disease, duration of diabetes, and timing of study entry were not available or not uniformly coded within the TriNetX network and could therefore not be included in the matching procedure. This limitation was addressed in the discussion section.

### 2.3 Outcome measures

The primary outcome was defined as “Fournier’s gangrene” (International Classification of Diseases [ICD-10] codes N76.80 [FG in females] and N49.80 [GF in males]). Furthermore, the index event was defined as “use of SGLT2i and inpatient encounter” (cohort I) and “inpatient encounter” (cohort II). The time window was not limited in terms of a specific end date, so all outcome events after the index event were recorded (as long as SGLT2i were still used, referring to cohort I). Outcome events were registered at a daily interval.

### 2.4 Data analysis

After matching, a risk analysis was performed, and measures of the association, including risk difference, risk ratio (RR) and odds ratio (OR) with their respective 95% confidence intervals (CI), were calculated. Statistical analysis was performed using the Log-Rank test, whereby p ≤ 0.05 was defined as the significancethreshold. As it was unknown since when the patients within cohort I had been using SGLT2i before the index event, all individuals having outcomes before the index event were excluded from the risk analysis.

## 3 Results

### 3.1 Cohort characteristics

**FIGURE 1:**
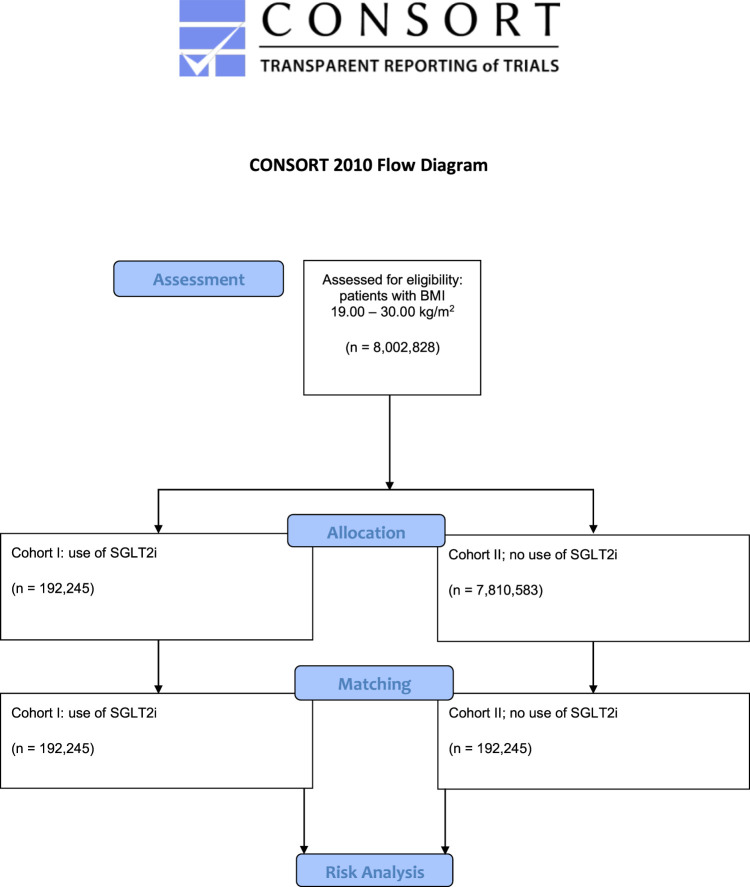
Modified Consolidated Standard of Reporting Trials (CONSORT) flow chart. The diagram illustrates the data extraction process from the TriNetX database. The total cohort size of n = 8,002,828 subjects consisted of 192,245 individuals using sodium-glucose cotransporter 2 inhibitors (SGLT2i) and 7,810,583 patients who had never been treated with SGLT2i. The database was accessed on 27 November 2024.

**FIGURE 2:**
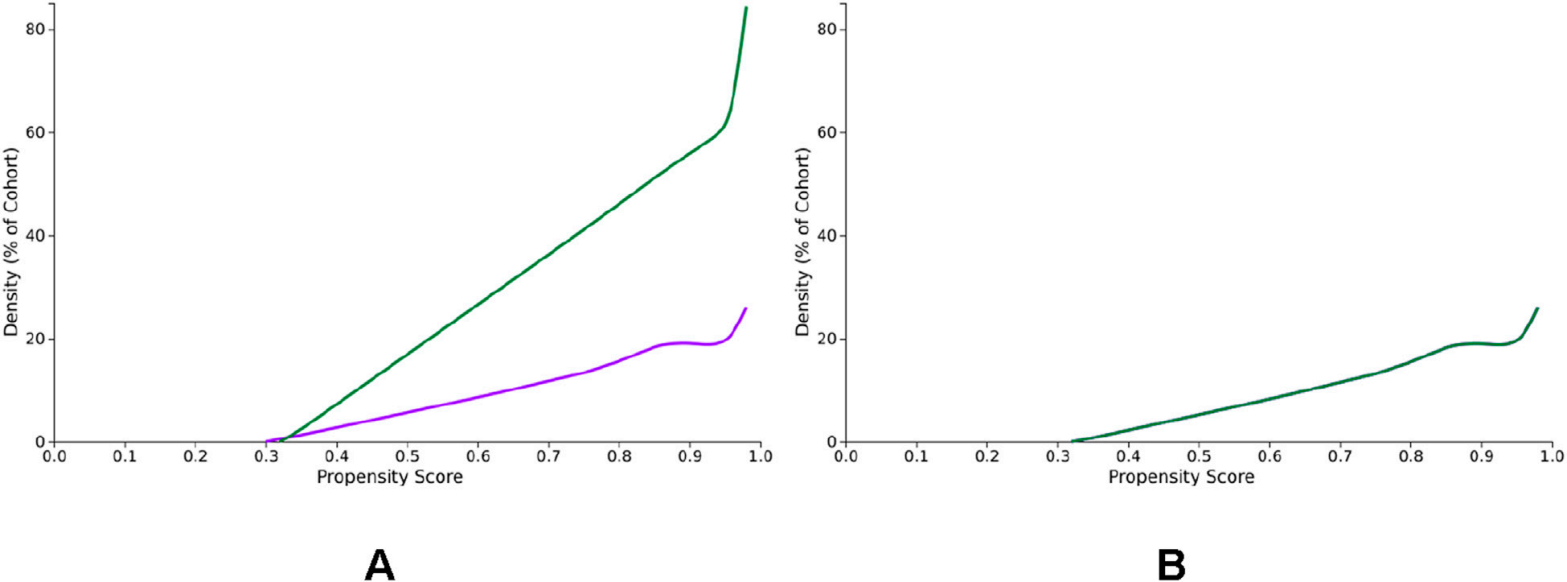
**(A,B)** Density of the cohorts I and II before and after propensity score matching for age, gender distribution, and prevalence of diabetes mellitus, human immunodeficiency virus infection, alcohol abuse, liver diseases, and use of immune suppressants. Cohort I (purple): patients using sodium-glucose cotransporter 2 inhibitors (SGLT2i) and cohort II (green): subjects not using SGLT2i.

The access date was 27 November 2024. Sixty HCOs provided medical records. Before matching, cohorts I and II accounted for 192,245 and 7,810,583 patients, respectively. After one-to-one propensity score matching, 192,245 subjects (40.5% females and 59.5% males; mean age 67.5 ± 12.7 years) remained in each cohort (Figure 1). The characteristics of the cohorts before and after matching are shown in Table 1. The density of the cohorts before and after matching is displayed in Figures 2A,B.

**TABLE 1:** Characteristics of cohorts I (use of sodium-glucose cotransporter 2 inhibitors [SGLT2i]) and II (no use of SGLT2i) before and after propensity score matching for age, gender distribution, and prevalence of diabetes mellitus, human immunodeficiency virus infection, alcohol abuse, liver diseases, and use of immune suppressants.

Cohort I (n = 192,245) and cohort II (n = 7,810,583) before propensity score matching						
Demographics						
Cohort		Mean ± SD	Patients	% of Cohort	p	Std. diff
I II	Age	67.5±12.7 52.0±22.3	192,245 7,676,831	100% 100%	<0.001	0.858
I II	Female		77,836 3,988,168	40.5% 52.0%	<0.001	0.332
I II	Male		114,409 3,822415	59.5% 48.0%	<0.001	0.312
Medical conditions						
I II	Diabetes mellitus		132,522 816,078	68.9% 10.6%	<0.001	1.483
I II	Human immunodeficiency virus (HIV) infection		2,564 35,723	1.3% 0.5%	<0.001	0.092
I II	Alcohol abuse		10,019 178,433	5.2% 2.3%	<0.001	0.152
I II	Diseases of liver		36,426 347,977	18.9% 4.5%	<0.001	0.459
Medication						
I II	Immune suppressants		9,816 113,344	5.1% 1.5%	<0.001	0.205

Cohort I (n = 192,245) and cohort II (n = 192,245) after propensity score matching						
Demographics						
Cohort		Mean ± SD	Patients	% of Cohort	p	Std. diff
I II	Age at index	67.5±12.7 67.5±12.7	192,245 192,245	100% 100%	0.941	<0.001
I II	Female		77,836 77,800	40.5% 40.5%	0.922	<0.001
I II	Male		114,409 114,445	59.5% 59.5%	0.906	<0.001
Medical conditions						
I II	Diabetes mellitus		132,521 132,531	68.9% 68.9%	0.972	<0.001
I II	Human immunodeficiency virus (HIV) infection		2,563 2,528	1.3% 1.3%	0.621	0.002
I II	Alcohol abuse		10,018 9,970	5.2% 5.2%	0.727	0.001
I II	Diseases of liver		36,425 36,432	18.9% 19.0%	0.977	<0.001
Medication						
I II	Immune suppressants		9,815 9,785	5.1% 5.1%	0.826	0.001

SD, standard deviation; Std. diff. = standardized difference.

### 3.2 Risk analysis

Among the cohorts I and II 186 and 312 individuals were diagnosed with FG after the index event, whereby 585 and 426 subjects were excluded from the risk analysis as they developed FG before the index event. The according risks of 0.2% and 0.1% were significantly different (p > 0.001; Log-Rank test). RR and OR were 0.539 (95% CI 0.447-0.650) and 0.539 (95% CI 0.446-0.650). The results of the risk analysis are summarized in Table 2.

**TABLE 2:** Analysis of the risk of developing Fournier’s gangrene in cohort I (patients using sodium-glucose cotransporter 2 inhibitors [SGLT2i]) and cohort II (individuals not using SGLT2i). Among the cohorts I and II 585 and 426 patients were not included into the risk analysis as the outcome occurred before the index event.

Risk analysis excluding patients with outcome prior to the time window			
Cohort	Patients included into risk analysis	Patients with outcome	Risk
I	191,660	168	0.1%
II	191,819	312	0.2%
		95% CI	p
Risk difference	0.1%		<0.001
Risk ratio	0.539	0.447-0.650	
Odds ratio	0.539	0.446-0.650	

## 4 Discussion

The study was set up assuming that no significant difference in the risk of FG could be found between patients using SGLT2i and those not using SGLT2i. The hypothesis was not confirmed. The performed analysis even revealed a lower risk in individuals using SGLT2i in comparison with patients not using SGLT2i. As this finding is not in accordance with the literature presented in the introduction we repeated the risk analysis including all cases of FG which were excluded from the initial analysis (585 and 416 patients from cohorts I and II) as outcomes had occurred before the index event (inpatient encounter). After performing a secondary calculation with a total number of 753 and 738 individuals with FG among the cohorts I and II, no statistically significant difference was detected regarding the risk of FG between both cohorts (p > 0.05). This finding is consistent with the recent literature (Silverii et al., 2020; Watada et al., 2020; Fisher et al., 2020). Furthermore, the confidence intervals obtained from the primary analysis support the result that the risk of FG does not differ between the cohorts. As a consequence, the initially obtained result needs to interpreted most cautiously, and it shows that further research is necessary.

In addition, specific limitations that come alongside with the applied study design need to be considered. Both cohorts were matched for the frequency of diabetes mellitus (68.9% of the patients among cohorts I and II after matching) because diabetes is known to be a risk factor for FG (Lewis et al., 2021). Thus, it may be hypothesized that the positive effects of SGLT2i on diabetes could have mitigated the risk of FG associated with the disease among the patients in cohort I, whereas the effect was absent in cohort II. This could explain the finding of our study. At least, this thesis might encourage further research focusing on metabolic control through SGLT2i use, specifically measurements of HbA1c values, and their influence on the risk of FG in diabetics. From TriNetX no respective data was available.

Another important limitation lies in the availability and consistency of certain clinical variables within the TriNetX database. While key confounders such as age, sex, and major comorbidities could be matched reliably, additional factors like chronic kidney disease, duration of diabetes, and the exact timing of study inclusion could not be integrated into the propensity score matching due to data heterogeneity and incomplete coding across participating healthcare organizations. This may have introduced residual confounding, which must be considered when interpreting the results.

In accordance with the FDA the authors suggest that healthcare professionals should frequently assess patients using SGLT2i for Fournier’s gangrene and if suspected, immediately initiate therapy with antibiotics and surgical debridement if indicated.

## Data Availability

The raw data supporting the conclusions of this article will be made available by the authors, without undue reservation.

## Abbreviations

AHF, acute heart failure; CI, confidence interval; CKD, chronic kidney disease; DPP4i, dipeptidyl peptidase-4 inhibitors; FDA, Food and Drug Administration; FG, Fournier’s gangrene; HF, heart failure; HIV, human immunodeficiency virus; MH-OR, Mantel-Heanzel odds ratio; OR, odds ratio; RR, risk ratio; SGLT2i, sodium-glucose cotransporter 2 inhibitors; T2DM, type 2 diabetes mellitus.
